# High resolution copy number inference in cancer using short-molecule nanopore sequencing

**DOI:** 10.1093/nar/gkab812

**Published:** 2021-09-22

**Authors:** Timour Baslan, Sam Kovaka, Fritz J Sedlazeck, Yanming Zhang, Robert Wappel, Sha Tian, Scott W Lowe, Sara Goodwin, Michael C Schatz

**Affiliations:** Cancer Biology and Genetics Program, Memorial Sloan Kettering Cancer Center, New York, NY, USA; Department of Computer Science, Johns Hopkins University, Baltimore, MD, USA; Human Genome Sequencing Center, Baylor College of Medicine, Houston, TX, USA; Cytogenetics Laboratory, Memorial Sloan Kettering Cancer Center, New York, NY, USA; Cold Spring Harbor Laboratory, Cold Spring Harbor, NY, USA; Cancer Biology and Genetics Program, Memorial Sloan Kettering Cancer Center, New York, NY, USA; Cancer Biology and Genetics Program, Memorial Sloan Kettering Cancer Center, New York, NY, USA; Howard Hughes Medical Institute, Chevy Chase, MD, USA; Cold Spring Harbor Laboratory, Cold Spring Harbor, NY, USA; Department of Computer Science, Johns Hopkins University, Baltimore, MD, USA; Cold Spring Harbor Laboratory, Cold Spring Harbor, NY, USA; Department of Biology, Johns Hopkins University, Baltimore, MD, USA

## Abstract

Genome copy number is an important source of genetic variation in health and disease. In cancer, Copy Number Alterations (CNAs) can be inferred from short-read sequencing data, enabling genomics-based precision oncology. Emerging Nanopore sequencing technologies offer the potential for broader clinical utility, for example in smaller hospitals, due to lower instrument cost, higher portability, and ease of use. Nonetheless, Nanopore sequencing devices are limited in the number of retrievable sequencing reads/molecules compared to short-read sequencing platforms, limiting CNA inference accuracy. To address this limitation, we targeted the sequencing of short-length DNA molecules loaded at optimized concentration in an effort to increase sequence read/molecule yield from a single nanopore run. We show that short-molecule nanopore sequencing reproducibly returns high read counts and allows high quality CNA inference. We demonstrate the clinical relevance of this approach by accurately inferring CNAs in acute myeloid leukemia samples. The data shows that, compared to traditional approaches such as chromosome analysis/cytogenetics, short molecule nanopore sequencing returns more sensitive, accurate copy number information in a cost effective and expeditious manner, including for multiplex samples. Our results provide a framework for short-molecule nanopore sequencing with applications in research and medicine, which includes but is not limited to, CNAs.

## INTRODUCTION

Copy number variation is a common form of genomic variation in humans and has been found to be correlated with a number of pathologies including rare genomic disorders (1), neurological diseases (2,3) and cancer (4). In cancer, somatically acquired Copy Number Alterations (hereafter reference to as CNAs) contribute substantially to the remodeling of cancer genomes with diagnostic, prognostic, and therapeutic implications (5,6). Traditionally, CNA information has been retrieved via methods such as chromosome analysis/karyotyping and DNA-FISH (7), which have limitations in sensitivity and/or resolution of analysis. Next generation sequencing addresses these limitations by showing that high resolution CNA information is retrievable from short-read sequencing data using a variety of experimental and computational approaches (8–13). These efforts have culminated in the successful implementation of short-read sequencing in the clinic (14,15). However, such applications are still associated with substantial costs and have been largely confined to large, resource rich, clinical centers. Enabling smaller, community-based centers with the ability to retrieve cancer sequence information, in a cost-effective manner, is likely to enhance the quality of health care overall.

New technological advances have paved the path for a newer form of sequencing termed nanopore sequencing (16,17). Nanopore sequencing relies on the detection of ionic current fluctuations as DNA molecules translocate through a protein pore (i.e. nanopore) and the subsequent transformation of these signals into nucleotide information (18). Nanopore sequencing offers many advantages compared to traditional short-read sequencing technologies, including single-molecule detection, portability, and low instrument cost as well as the ability to retrieve sequence information from large contiguous fragments of DNA, i.e. long read sequencing (19). Of these advantages, the ability to sequence long DNA molecules has received the most attention in biomedical research because of its potential to shed light on complex areas of the genome, such as repetitive elements and segmental duplications, areas that remain largely unexplored (20). Further, long read nanopore sequencing has been shown to reveal cryptic genetic variation that is missed by traditional short-read sequencing approaches, accentuating the importance of this emerging sequencing technology in biomedical research and, potentially, clinical practice (21–23).

One major shortcoming of nanopore sequencing has been the relatively low yield in terms of the number of distinct sequenced DNA molecules. For example, compared to an Illumina NextSeq machine run, which typically returns hundreds of millions of short-reads, a standard long DNA molecule nanopore run on a MinION device yields an average of approximately one million reads. This limitation has hindered wide-spread utility of nanopore sequencing in research and clinical sequencing applications that require high read counts, especially CNA inference. Here, we apply a simple yet effective experimental approach to address this limitation by loading and sequencing relatively short molecules of DNA and show that it reproducibly returns high read/molecule counts, sufficient for high resolution CNA analysis using the widely studied SK-BR-3 breast cancer cell line as well as seven acute myeloid leukemia (AML) samples. Our approach provides a foundation for future studies where real-time, point of care sequencing applications in cancer, and other diseases, can be done with minimal infrastructure in clinical centers, large and small alike.

## MATERIALS AND METHODS

### Samples included in study, DNA extraction, and sequencing library preparation

To develop the short molecule sequencing approach, we first experimentally tested DNA library preparation conditions and sequencing on DNA retrieved from a widely studied breast cancer cell line, SK-BR-3, which we and others have used as a reference for CNA method development (9,24,25). For nanopore sequencing library preparation, SK-BR-3 cell line DNA was fragmented to ∼500 bp using the Covaris Instrument (Woburn, MA). 300 ng of sonicated material was A-tailed and end repaired using the NEB A-tailing end repair module. Adaptor ligation for nanopore sequencing was performed using the SQK-LSK-109 kit from Oxford Nanopore. Three different DNA library loading masses were tested to determine optimal loading condition; 15, 45 and 90 ng. 15 ng is equivalent to 50 fmol of dsDNA; the maximum, manufacturer suggested mass gDNA for loading. We hypothesized that a larger mass of DNA could improve throughput for smaller fragments, thus 45 ng (3× the suggested molar mass) and 90ng (6x the molar mass) were also tested. Each aliquot of library was loaded onto a R9.4.1 MinION flow cell and allowed to run for 48 h on a GridION.

For clinical implementation feasibility studies, we relied on sequencing DNA retrieved from acute myeloid leukemia (AML) samples. Five samples were considered for MinION sequencing, two that were cytogenetically classified as normal karyotype and three classified as complex karyotype (CK-AML), with the latter known to confer a dismal prognosis in AML disease. We also sequenced two additional AML samples, one complex and one normal, using the low throughput Flongle. DNA was extracted from bone marrow leukemic blasts using a Qiagen AllPrep DNA/RNA Mini Kit following recommended manufacturer's protocols. Purified DNA was similarly processed for nanopore sequencing as per final experimental conditions established using SK-BR-3 DNA. For multiplex nanopore sequencing, DNA was prepared as described above with the following exception: after the initial A-tailing and end repair step, DNA was ligated to a PCR based barcoding adapter (EXP-PCB096) for unique indexing with a hamming distance of at least 10 between their sequences. The sequences for the indexes are as follows: BC01/RB01: AAGAAAGTTGTCGGTGTCTTTGTG, BC02/RB02: TCGATTCCGTTTGTAGTCGTCTGT, BC03/RB03: GAGTCTTGTGTCCCAGTTACCAGG, BC04/RB04: TTCGGATTCTATCGTGTTTCCCTA, BC05/RB05: CTTGTCCAGGGTTTGTGTAACCTT. Five barcoded samples were then pooled in an equimolar fashion, with a total mass of 300ng between all five samples. The pooled samples were processed with the SQK-LSK-109 kit. Samples were base called with the on-board GPU system with Guppy v3.1. The research involving human subjects, specifically the AML samples, was approved by the authors Institutional Review Board (MSKCC IRB).

For each analyzed sample (SK-BR-3 as well as AML samples), a matching short-read Illumina sequencing library was constructed and sequenced on a HiSeq device using standard Illumina protocols (San Diego, CA).

### Nanopore sequence read base calling, processing, and analysis

To ascertain an appropriate short DNA molecule length to sequence on a Nanopore device, we performed an analysis where long molecule nanopore sequencing data of at least 10 kb were computationally truncated to shorter molecules that differed by 100 bp, starting from 100 bp and ending with 1 kb (e.g. 10 different read length classes). All reads were mapped to the hg38 genome built, excluding alternate scaffolds. MAPQ is defined as −10 × log_10_ Pr(alignment is wrong), and minimap2 reports a maximum MAPQ value of 60. The reads were partitioned into bins based on read length, and within each bin we computed the fraction of unaligned reads, reads with perfect MAPQ, and reads with sub-optimal MAPQ (Supplemental Figure S1A).

For each sequencing run we computed read length distributions, read counts over time, channel lifetimes, molecule residence time (i.e. the time duration for an actively sequenced read), pore vacancy time (i.e. the time between reads in a given channel), and channel activity. All summary statistics provided in Table 1 based on the ‘sequencing summary’ files output by the Guppy basecaller. These summary files include the time that each read started and finished sequencing, which channel reads came from, read lengths, and quality scores. Read counts were computed using a simple cumulative sum over the course of each run. ‘Relative Reads per Channel’ was computed by dividing the reads per channel by the number of channels on the device (512 for MinION, 126 for Flongle). Channel lifetimes were estimated by the time that each channel finished sequencing its final read. The distribution of vacancy time between reads was computed by subtracting the start and end times of consecutive reads, excluding reads which were not sequenced by the same pore within a channel. The channel activity plots were generated by dividing each channel into five minute windows and coloring a window black if the channel sequenced any length of a read during that time.

**Table 1.** Annotations and sequencing statistics for samples run on a MinION device

Sample/type	Sample ID	Read count	Yield (Mb)	Passed (%)	*Q*-score mean	Read-length mean	Read N50	Minutes to 250k reads
Cancer Cell Line SKBR3	SKBR3-Long	899425	8360.86	84.94	10.17	9295.79	14301	291.93
Cancer Cell Line SKBR3	SKBR3-Short (1ME)	6332668	3024.8	86.42	10.54	477.65	538	16.5
Cancer Cell Line SKBR3	SKBR3-Short (3ME)	1465247	789.33	89.61	10.89	538.7	637	17.07
Cancer Cell Line SKBR3	SKBR3-Short (6ME)	699009	416.13	88.69	10.73	595.32	732	19.35
Leukemia (AML-BM)	AML1	3797811	1670.22	84.97	9.09	439.79	501	15.13
Leukemia (AML-BM)	AML2	6065542	2510.64	81.17	8.69	413.92	461	16.42
Leukemia (AML-BM)	AML3	2312000	1072.69	91.29	9.72	463.96	503	16.59
Leukemia (AML-BM)	AML4	3935726	1753.57	86.68	9.29	445.55	488	15.58
Leukemia (AML-BM)	AML5	6263490	2886.35	82.82	8.95	460.82	519	15.53

AML = acute myeloid leukemia, ME = molar equivalent.

All AML samples were run at 1ME concentrations.

### Copy number inference from short read and short molecule sequencing data

Nanopore sequencing data was mapped to human reference genome hg19 using Minimap2 (26) software with the following parameters: -ax map-ont -t 15. Uniquely mapped reads were subsequently filtered using Samtools (27) with the following parameters: -Sbu -q 20 -F 0 × 904. Sorted reads were indexed and counted in genomic bins of median length of 600 000 and 150 000 kb derived by dividing the genome into the five thousand (5k) and twenty thousand (20k) bins respectively, using the Varbin algorithm (25,28). Normalized bin counts were subsequently segmented using Circular Binary Segmentation (29) with stringent segmentation requiring five contiguous bins to call a copy number transition. Thus, at 5k and 20k bin resolutions, the analysis can segment and detect copy number events at resolutions of 3 Mb (5 × 600 000 kb) and 0.75 Mb (5 × 150 000 kb). Segmented read count data were subsequently transformed to absolute copy number values using a least square fitting algorithm (9,24). Short read Illumina sequencing data was sequenced at depths comparable to short molecule nanopore data (median depths of ∼ 8 million total reads per sample) and processed as previously described (24).

### AML sample chromosomal analysis

Fresh bone marrow aspirates were used for conventional chromosome analysis following standard protocol, without mitogen stimulation in short term culturing. At least 20 metaphase cells were analyzed, and karyotype was described according to the international system of human chromosome nomenclature (7). Independent numerical and structural chromosomal abnormalities, such as gain, loss, deletion, addition, duplication, balanced or unbalanced translocations, inversion and derivative chromosomes, as well as marker chromosome, ring chromosome were annotated in defining complex karyotypes, i.e. three or more, clonal heterogeneity, and monosomal karyotype.

### DNA-FISH validation of identified copy number alterations in AML

FISH analysis was performed on bone marrow or peripheral blood pellets from cytogenetic analysis in selected samples, following standard protocols. Various commercial FISH probes, specific for myeloid neoplasia, such as for deletion or loss of chromosomes 5, 7, 17, gain of chromosome 8, and for MLL/KMT2A (11q23) translocations, EVI1 (3q26.2), and other translocations, were used as appropriate. All probes were purchased from several companies, such as Abbott Molecular (Des Plaines, IL), Metasystems (Newton, MA). At least 200 cells were analyzed, and results were described according to (7). In all samples, correlation of FISH results and chromosome analysis findings were also compared.

## RESULTS

### Sequencing short DNA molecules on a nanopore device yields high read counts and enables accurate copy number profiling

The number of reads/molecules returned in a standard long read MinION run is limited by (i) the design of the nanopore array, especially the overall number of channels and the overall number of active pores; (ii) the molecule kinetics of DNA fragments docking to the nanopore, measured by the duration between the end of one read and the beginning of the next within the same pore (the vacancy time) and (iii) the residence time of a molecule translocating through a given pore. Consequently, we hypothesized that loading DNA molecules that are shorter in length (e.g. 400 bp in median length compared to a standard 10 kb) would decrease the residence time of each DNA molecule in a pore and thus facilitate, over a period of time, the translocation of more DNA molecules and the retrieval of higher read/molecule counts.

To experimentally test our hypothesis, we sonicated DNA purified from the SK-BR-3 breast cancer cell line to an average length of 400 bps and prepared a nanopore sequencing library using standard protocols (Materials and Methods). We targeted an average DNA molecule length of 400 bps in part due the relationship between length and mappability of DNA molecules on a nanopore device (Supplementary Figure S1A, Materials and Methods) and to allow direct comparisons with Illumina sequencing data. For short read/molecule sequencing on a R9.4.1 MinION flowcell, we decided to load libraries at concentrations of 1, 3 and 6× the Molar Equivalent (ME) of standard loading conditions (Materials and Methods), henceforth referred to as Short-1ME, Short-3ME and Short-6ME respectively. Varying the loading conditions we performed to ascertain the relationship between number of molecules loaded and subsequently sequenced in each experiment. As a control, a long read sequencing library from the same SK-BR-3 DNA was generated and sequenced using standard protocols (Materials and Methods).

**Figure 1.**
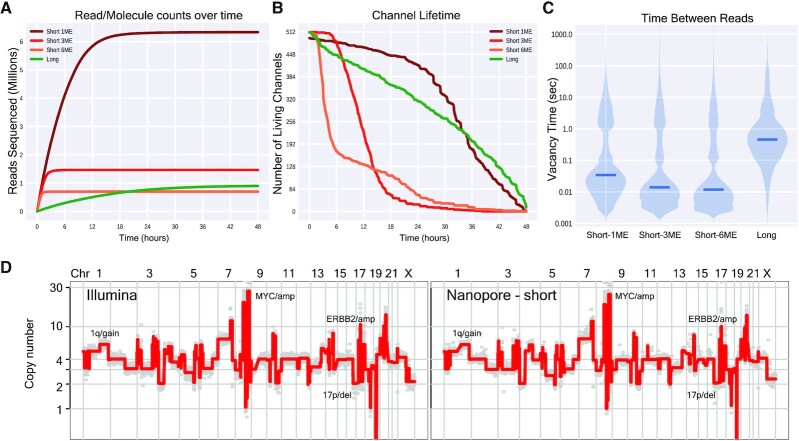
Sequencing short molecules on a nanopore device yields high read counts and enables accurate copy number profiling. (**A**) Cumulative number of reads/molecules sequenced over time throughout each SK-BR-3 nanopore run (ME = molar equivalent). (**B**) Total number of ‘living’ channels over time throughout each run as measured by the time that the last read was sequenced by each channel. Note that a channel may not produce a read for several hours but still be considered ‘alive’ by this definition. (**C**) Distributions of timing between discrete DNA molecules entry/exit from a given channel, i.e. vacancy time. (**D**) Genome-wide copy number profiles of SK-BR-3 sequenced using short molecule nanopore (right panel) and short-read Illumina (left) data. Profiles are plotted when dividing the genomes in 5 thousand bins (i.e. 5k bins). Examples of detected CNAs are annotated on the profiles.

Sequencing the long molecule nanopore library resulted in an expected yield of ∼1 million molecules. By contrast, loading the Short-1ME sequencing library resulted in roughly 6 million sequenced molecules, a 6-fold increase (Figure 1A). Interestingly, while the number of active sequencing channels decayed with a similar trend for both sequencing conditions (Figure 1B), the increased read yield in the Short-1ME library was largely attributed to an accelerated return of sequenced molecules over the first 12 hours (Figure 1A, Supplementary Figure S1B). We attribute the increase in sequenced molecule return primarily to reduced residency and vacancy time. Residency time is directly proportional to the length of the molecules and the speed of sequencing, for example ∼1 s for a 400 bp molecule compared to 22 s for a 10 kb molecule when sequencing at 450 bp per second. Further, vacancy time is also shorter when sequencing short molecules, regardless of loading concentration (Figure 1C). Short-1ME sequencing reads were of similar quality to long reads when comparing sequencing metrics such as Pass Filter (86% Short-1ME versus. 85% Long) and mean Q-Score values (10.54 Short 1ME versus 10.17 Long) (Table 1). The average length of the sequenced DNA molecules was confirmed to be roughly 500bps (Supplementary Figure S1C).

Importantly, following normalization (Methods and Supplementary Figure S2), processing of the data for copy number inference using previously developed algorithms (9,24) that relate read depth (i.e. molecules sequenced at a given genome locus) to genome copy number, the data returned highly accurate copy number information at various resolutions compared to information retrieved from standard Illumina short-read sequencing as well as long molecule nanopore data (Materials and Methods) (Figure 1D and Supplementary Figure S3A). Detected copy number events ranged in size from a few megabases to whole chromosome arm and chromosome level events (Figure 1D and Supplementary Figure S3B). These included: (i) focal amplifications at the *MYC* and *ERBB2* genes, (ii) gain on 1q and (iii) deletion on 17p, all CNAs of variable sizes shown to be associated with a poor prognosis across a range of human tumors. Furthermore, read count downsampling analysis revealed consistently accurate genome-wide copy number inference at depths of 2 million, 1 million, and 500 000 reads total, consistent with prior published work. Downsampling to 250 000 total reads and below frequently resulted in spurious segments and erroneous copy number calls (Supplementary Figure S3C).

Short-3ME and Short-6ME libraries yielded 11 and 6 times more sequenced reads respectively relative to long reads over the first two hours, however the sequencing yield pattern decayed rapidly (Figure 1A). This pattern was associated with a diminishing number of active channels over time as well as vacancy time relative to the Short-1ME sequencing experiment (Figure 1A–C). As the quality of the data is similar to that from long and Short-1ME data (Table 1, Supplementary Figure S1B), we hypothesize that this decline may be a consequence of the mechanics of nanopore/electrical circuitry and/or limiting reagent availability to drive the translocation of DNA molecules through the MinION nanopore channels.

Overall, these data show that loading short DNA molecules, at an appropriate concentration, to a MinION nanopore device can yield up to 6 million sequenced molecules in a single run, data which can be used to infer CNAs with high accuracy.

### Short molecule sequencing on a MinION device allows accurate, high resolution copy number inference in a clinically relevant setting

To assess the potential utility of short molecule sequencing on MinION devices in retrieving clinically actionable and relevant information, we turned to the analysis of acute myeloid leukemia (AML) genomes where extensive evidence exists on the prognostic importance of CNAs (i.e. karyotypes/cytogenetics). Five samples were sequenced, two that were cytogenetically classified as normal karyotype and three classified as complex karyotype (CK-AML), with the latter known to confer a dismal prognosis in AML disease. All AML samples were sequenced using Short-1ME loading conditions. In parallel, all samples were sequenced on an Illumina machine for comparative analysis.

**Figure 2.**
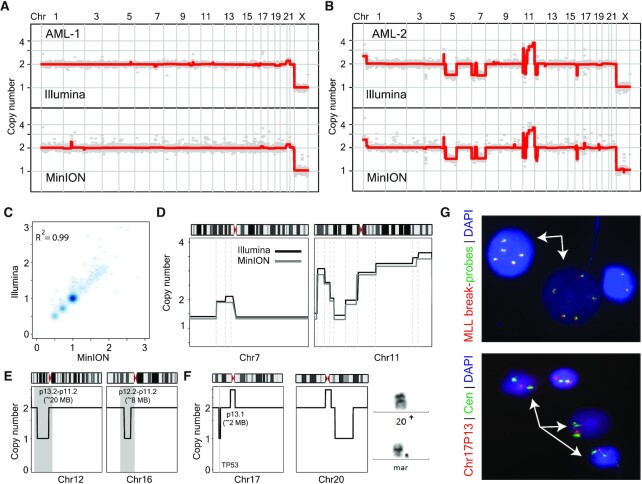
Short molecule sequencing on a MinION nanopore device yields accurate high resolution copy number information in a clinically relevant setting. (**A**) and (**B**) Genome-wide copy number profiles of a normal and a complex karyotype sample sequenced using short molecules on a MinION (lower panel) and short-read on an Illumina instrument (upper panel). (**C**) Density scatter correlation plot of normalized bin read counts (proportional to copy number) from MinION and Illumina sequencing data for leukemic sample AML-2. Pearson correlation value is provided. (**D**) Zoom in views of chromosome 7 (left panel) with complex rearrangement resulting in loss of both short and long arms and chromosome 11 (right panel) with gains of the long arm at various level in complex karyotype sample AML-2 using Nanopore (gray line) and Illumina (black line) sequencing data. Dashed gray vertical lines illustrate alteration breakpoints. (**E** and **F**) Zoom in views of chromosomal deletions on chromosomes 12p, 16p, 17p and 20q. Right panel in (**F**) illustrates chromosome images derived from cytogenetic analysis showing a normal chromosome 20 (top) and a marker chromosome (bottom) which was redefined as a derivative chromosome 20 with deletion of the long arm based on this study (**G**) DNA-FISH based validation of selected CNA alterations. Gain of the MLL gene on chromosome cytoband 11q23 (top panel), using a break apart MLL probe set (5’MLL and 3’MLL are labeled in orange and green respectively); two cells (arrows) show five copies of MLL/KMT2A. FISH tests confirm focal 17p loss, encompassing the TP53 gene (bottom panel). TP53 probe and D17Z1 (CEP17-Cen) probes are labeled in orange and green, respectively. Three cells (arrows) show two green signals for CEP17 and only one orange signal for TP53. Nuclei are counterstained with DAPI (blue).

All AML samples sequenced using Short-1ME loading conditions yielded expected high read counts (∼4–6 million reads per sample) (Table 1). Further, all samples displayed similar high-quality sequencing statistics, such as Pass Filter and mean Q-Scores, as with the Short1-ME SK-BR-3 data (Table 1). When processed for copy number inference, we find that the nanopore Short-1ME data yields largely similar genome-wide profiles and CNAs with Pearson correlation coefficients above 0.98 across all samples when compared to Illumina data (Figure 2A–C and Supplementary Figure S4). For example, for sample AML2, nanopore sequence data inferred CNAs that were highly correlated to those inferred from Illumina sequencing data (Pearson correlation values > 0.99) with large deletions on chromosomes 5 and 7 (over 10MBs) as well as complex re-arrangements on chromosome 11 resulting in *MLL1/KMT2A* gene gains identified. Further, identified alterations shared largely identical breakpoint positions in both datasets (Figure 2B–D). Moreover, focal alterations on chromosome arms 12p and 16p were also identified in both nanopore and Illumina sequencing data and corroborated with standard cytogenetic analysis (Figure 2E and Supplementary Figure S4).

In multiple cases, nanopore sequencing data resulted in more accurate copy number information compared to standard cytogenetic chromosome analysis. For example, in AML5, cytogenetically reported monosomy (i.e. loss of an entire chromosome) for chromosome 20 was shown in both Illumina and nanopore sequencing data to be inaccurate, most likely the result of incorporation of chromosome 20 genetic material into marker chromosomes (Figure 2F). Likewise, copy number analysis at higher resolution, afforded as a direct consequence of the increased read counts, identified a focal deletion on the short arm of chromosome 17 (17p) encompassing the *TP53* gene in the sequencing data (Figure 2F). Selected alterations (*MLL* and *TP53*) were validated using DNA-FISH analysis in their respective samples (Figure 2G).

Together, these results show that short molecule sequencing on a MinION device reproducibly yields high read/molecule counts that are of high quality. Further, when processed informatically, the data results in highly accurate copy number profiles that are concordant with both matching Illumina data and standard cytogenetic results. The data also show that short molecule nanopore sequencing returns cryptic CNA information that is either missed or mis-classified in standard cytogenetic, chromosome-based analyses.

### High read counts enable multiplex sequencing and copy number inference of AML samples on a MinION device

Sequencing-based copy number inference tools can rely on sparse, low coverage sequencing to relate sequencing depth in genomic intervals to copy number (9,24). Furthermore, sequencing depth can be adjusted to accommodate varying resolution of analysis, typically targeting an average of 30–50 reads per genomic bin for a reliable CNA inference (9,24). Given the comparatively high number of sequencing molecules retrieved by sequencing Short-1ME nanopore libraries, multiplexing of different samples on the same MinION run is theoretically possible. Multiplexing would lower the costs associated with retrieving sequencing-based copy number information as well as provide flexibility in terms of sample processing, both issues that are relevant from a clinical implementation perspective. To this end, we generated uniquely indexed short molecule nanopore sequencing libraries for all five sequenced AML samples. Indexed AML samples were subsequently pooled at equal molar concentrations and the resulting pool sequenced on a single MinION device run (Materials and Methods).

**Figure 3.**
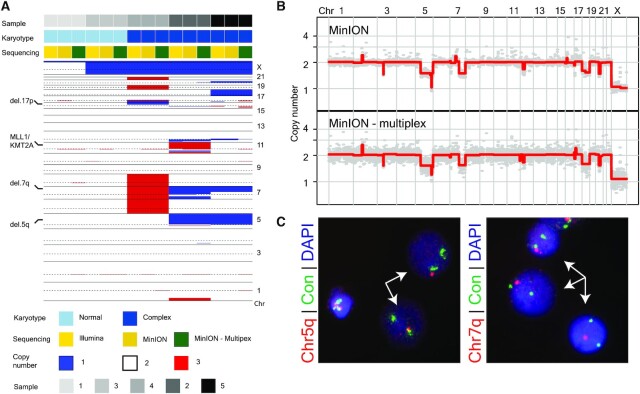
Increased read counts via short molecule sequencing enables accurate, multiplex profiling on a MinION device. (**A**) Heatmap illustration of copy number profiles of all profiled AML samples (*n* = 5) using Illumina, MinION, and Multiplex MinION sequencing. Annotations regarding karyotype status of leukemic sample (normal vs. complex) and sequencing modality are denoted on bars on top of the heatmap. Heatmap color and bar color codes are provided below the heatmap. Selected, prognostically relevant CNAs are identified on heatmap. (**B**) Genome-wide copy number profiles from a complex karyotype AML sample inferred from short molecule nanopore sequencing in multiplex (lower panel) and non-multiplex mode (upper-panel). (**C**) DNA-FISH based validation of identified copy number alterations on the long arm of chromosomes 5 and 7(Chr5q and Chr7q respectively). A 5p15.2 probe (labeled in green) and a centromeric probes (D7Z1, CEP7) for chromosome 7 were used as an internal control (Con). Left: two cells (arrow) show two green signals for 5p and only one orange signal for 5q. Right: all three cells (arrow) show two green signals for CEP7 and only one orange 7q. Color codes of probes are illustrated. Nuclei are counterstained with DAPI.

Pooled AML Nanopore run yielded slightly fewer number of sequenced molecules compared to previous short nanopore runs; ∼3.2 million reads. Regardless, the sequencing data was of high quality (93% passed filter and 11.15 mean *Q*-score) enabling 91% of the sequenced molecules to be assigned to a sample barcode (average of ∼600 000 reads per sample, 18% of total reads per sample) (Supplementary Figure S5A). When processed for CNA analysis, the multiplex data generated largely similar genome-wide copy number profiles across all samples compared to matching non-multiplex data, with Pearson correlation coefficients above 0.95 (Figure 3A, B and Supplementary Figure S5B–E). Identified CNAs, which were similarly detected in non-multiplex nanopore and Illumina sequencing data, included both deletions as well as gains, either as whole chromosome or interstitial, focal alterations, with events targeting important AML genomic regions such as 5q, 7q and 17p (Figure 3A, B and Supplementary Figure S5). Events were confirmed orthogonally using DNA-FISH experiments (Figure 3C).

Thus, the increase in return in terms of the number of high quality sequencing molecules afforded by loading short DNA fragments on a nanopore device facilitates multiplex sequencing and accurate inference of copy number information in an affordable and versatile manner.

### Short molecule sequencing using a Flongle adaptor yields high read counts and allows cheap portable sequencing

The MinION device has generally been known as a single-use nanopore sequencing device. Recently, Oxford Nanopore introduced the Flongle adaptor; a low-cost replaceable apparatus that utilizes similar chemistry in producing single molecule sequence data. This, in effect, makes the MinION device a repeat-use sequencing device. High read-count recovery via short molecule sequencing using a Flongle device theoretically can enable the retrieval of clinically actionable CNA data (i.e. karyotypes and CNA information) in settings outside large health care centers as well as countries with poorly developed health care infrastructure. To assess the feasibility of such applications, we generated short molecule nanopore libraries for a normal karyotype AML case and a complex karyotype AML case and using a Flongle adaptor for each, sequenced both. Short-read Illumina sequencing was also performed on DNA available for both cases.

**Figure 4.**
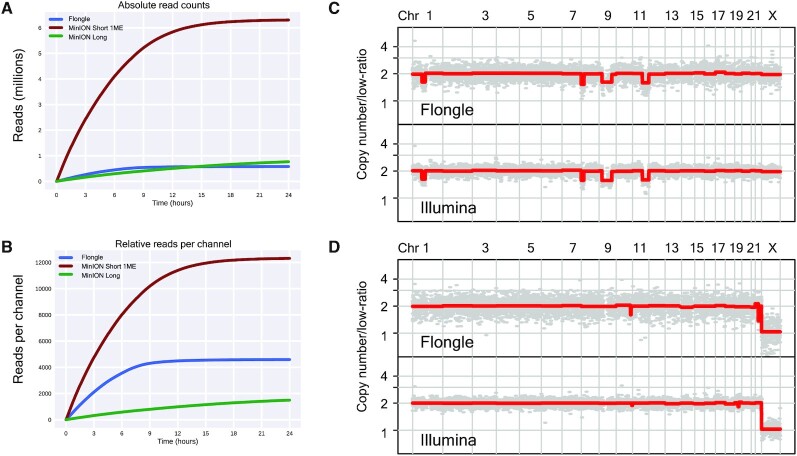
Short molecule sequencing using a Flongle adaptor enables high read counts, accurate copy number data, and cheap, portable sequencing. (**A**) Absolute number of sequenced molecules for a Flongle run compared to other nanopore runs. Short 1ME and Long are as depicted in Figure 1A. (**B**) Normalized number of sequenced molecules per channel for each nanopore run (126 channels for Flongle and 512 channels for MinION). (C and D) Genome-wide copy number profiles of a complex karyotype (**C**) and a normal karyotype (**D**) AML case inferred from short molecule nanopore sequencing (top-panel) and short read Illumina sequencing (lower panel), respectively.

Similar to short molecule sequencing on a non-Flongle MinION unit, Flongle based sequencing continued to return relatively high, short molecule sequence information over the first 12 h of the sequencing run with diminishing returns thereafter (Figure 4A and Supplementary Figure S6A). Short read Flongle runs yielded much lower numbers of sequenced molecules in terms of absolute counts; ∼600k and 300k for Flongle runs compared to ∼4–6 million MinION runs (Supplementary Figure S6B). However, when analyzing for number of sequenced molecules while normalizing for the number of sequencing channels (Flongle contains 126 channels compared to the standard 512 for a non-Flongle MinION unit), the data illustrate that Flongle short molecule sequencing returns almost one order of magnitude more data than a long molecule run (Figure 4B). The pattern of sequenced molecule return was similarly associated with a shorter vacancy time compared to long molecule sequencing, although vacancy time was longer than that observed with non-Flongle MinION Short-1ME runs (Supplementary Figure S6C). While Flongle runs yielded lower statistics for passed and mean *Q*-score (Supplementary Figure S6B), approximately 176 000 and 327 000 reads (83% and 86% of total pass filter reads) were uniquely mapped, enough to process for copy number analysis. Analysis of the Flongle nanopore data revealed that while one sample exhibited a normal copy number profile, the other sample displayed a pattern of deletions on chromosomes 1, 8, 9 and 11 resulting in complex karyotype annotations, consistent with matching cytogenetic data (Figure 4C and D).

Together, the data shows that the Flongle adaptor, when coupled with short molecule sequencing, is capable of facilitating sequencing applications that require a discrete number of sequenced molecules (ex: copy number profiling at specific resolutions). Furthermore, given the early stage nature of the Flongle in terms of development, we expect further modifications/improvements to yield more data within the context of short molecule sequencing in future iterations, and in doing so, allow higher resolution copy number analysis.

## DISCUSSION

Nanopore single molecule sequencing offers unique advantages compared to Illumina short read sequencing including real-time sequencing, portable sequencing, and the potential to sequence long molecules of DNA. Numerous studies have now shown the utility of each of the aforementioned advantages in the rapid re-identification of human samples, facilitating fast point-of-care diagnostics for infectious diseases, the first complete, telomere-to-telomere sequence of a human chromosome and several other applications (30–33). However, an important limitation of nanopore sequencing continues to be the relatively low number of sequencing reads returned per sequencing run, regardless of instrument/device (i.e. MinION, GridION or PromethION). This presents challenges for nanopore sequencing utility in ‘counting’ applications such as allelic genotyping/single nucleotide discovery and genome copy number inference, applications that require high read counts, at specified loci or distributed over a given genome, respectively. This is important as methods for the identification of CNAs, particularly hematological malignancies, have long been considered important tests/procedures to perform in the course of the clinical management of patients (5). Furthermore, increasing evidence has emerged regarding the importance of CNAs in more common, solid malignancies such as breast and lung cancer (6,34).

Here, by (i) making a simple optimization with regards to molecule length when factoring DNA molecule kinetics during translocation through a nanopore and (ii) applying an elementary strategy of loading short DNA molecules on a MinION device, we devise and validate a simple yet effective solution: short molecule nanopore sequencing. Loading short molecules of DNA (median length of 0.5 kb) on a MinION device consistently resulted in a 4- to 6-fold increase in the return of sequenced single molecules with the inferred CNA information concordant with that obtained from standard short-read Illumina sequencing and at much higher resolution than clinical karyotyping. Importantly, the increase in sequence molecule return on a nanopore run facilitated multiplex sequencing of clinical samples which decreases costs associated with sequence based copy number determination/karyotyping. Furthermore, in a multiplex sequencing setting, the increase in sequence molecule counts affords the flexibility to modulate sequence read counts to accommodate low purity samples or higher resolution analysis (e.g. for low purity sample or higher resolution analysis, more limited multiplexing and increased sequencing depths would be more advantageous). While the utility of Illumina short-read sequencing platform in retrieving CNA information in a clinical setting is useful and will garner broad application, our results show that CNA inference from short molecule nanopore sequencing provides a complementary platform associated with unique advantages. For example, CNA information from short molecule nanopore sequencing can easily be implemented in outreach clinical settings which are not equipped with Illumina machines and associated infrastructure. Supplementary Table S1 summarizes the advantages/disadvantages of both Illumina short-read and nanopore short molecule sequence based copy number detection.

The short molecule nanopore sequencing approach presented here has broad applications in research and clinical practice including, but not limited to, global DNA modification profiling via shotgun DNA sequencing as well as multiplex cancer gene amplicon sequencing. Most importantly, we show here that high molecule return on a nanopore can enable multiplex sequencing, that when coupled with molecular barcoding, vastly decreases the costs and time associated with the retrieval of clinically important information, such as karyotype information in AML disease. Additionally, in relation to sequence associated costs and potential clinical utility, we present results of short molecule nanopore sequencing within the context of the Flongle adaptor, providing further evidence for the potential for cost effective, point-of-care diagnostics using nanopore sequencing technology in cancer.

While others have devised innovative approaches using nanopore sequencing to address genomic applications that require high molecule/read counts (35), our approach is much simpler, especially for barcoded multiplexed samples. Hence, we surmise that short molecule nanopore sequencing is more likely to gain broad applicability and equally important, empower further technological developments in the rapidly evolving field of single-molecule sequencing. For example, we have recently reported on the development of an algorithm termed UNCALLED for the targeted enrichment of genomic sequences based on analysis of raw electrical nanopore signals and ejection of unwanted molecules from any given pore (36). Coupling the methods used in short molecule sequencing with targeted enrichment of specific loci could enable the retrieval of the entire repertoire of germline and somatic alterations found in a cancer, including single nucleotide variants, insertions and deletions as well as balanced structural variants all from a single sequencing run. Indeed, our early results point towards the feasibility of such an approach.

In conclusion, we show that short molecule nanopore sequencing facilitates increased sequence molecule return, enabling applications that require sequencing counts, such as CNA detection, that are inaccessible with standard, current nanopore practices (i.e. long molecule sequencing). We show that the data is high quality and when applied to address a biomedical question, such as retrieving clinically meaningful copy number information, returns accurate data at low costs and much flexibility in operation. We posit that short molecule nanopore sequencing will have broad applications, in DNA as well as RNA sequencing, that extend beyond genome copy number quantification.

## DATA AVAILABILITY

Raw data generated for this study are available through Short Read Archive (SRA) under BioProject accession number PRJNA693260. All code and reference files used in the described analysis are available from Baslan *et al.* (28). Additional code is available at Gingko (24), a web tool for analyzing copy number data (http://qb.cshl.edu/ginkgo/).

## Supplementary Material

gkab812_Supplemental_File
