# Supplementary material for: High resolution copy number inference in cancer using short-molecule nanopore sequencing
Source: Nucleic Acids Res. 2021 Sep 22;49(21):e124. doi: 10.1093/nar/gkab812 (PMC8643650; doi:10.1093/nar/gkab812)
Supplement: gkab812_Supplemental_File [file gkab812_supplemental_file.pdf]

Supplemental Information for
High resolution copy number inference in cancer using short-molecule nanopore sequencing

Timour Baslan[1*†], Sam Kovaka[2*], Fritz J. Sedlazeck[3], Yanming Zhang[4], Robert Wappel[5], Sha Tian[1], Scott W. Lowe[1,6], Sara Goodwin[5], Michael C. Schatz[2,5,7,†]

Table S1. Overview of short-read Illumina and short-molecule Oxford Nanopore sequencing for Copy Number Alteration Analysis.

Fig S1. Short DNA molecule sequencing on a MinION device yields a high number of sequencing reads.

Fig S2. Nanopore sequence data normalization and copy number inference in SK-BR-3 datasets.

Fig S3. MinION short molecule sequencing data returns highly accurate genome-wide copy number information.

Fig S4. Short molecule sequencing on a MinION device reproducibly yields accurate, high resolution copy number information.

Fig S5. Multiplex short molecule sequencing on a MinION device allows accurate and cheap inference of CNAs.

Fig S6. Short molecule sequencing using a Flongle adaptor returns high read counts and allows accurate copy number inference.

Supplementary Table 1. Overview of short-read Illumina and short-molecule Oxford Nanopore sequencing for Copy Number Alteration Analysis.

Instrument/Quality	Cost.Instrument ($)	Cost.reagent.per.run ($)	Yield.Variability	Portability (Weight)***	Time.to.500k.reads (hours)****	Total.read*****	multiplex.per.run (n)	Sequencing.cost ($)******
NextSeq	High (355k)	1.2k	Low	163 kg	24	130	High (260)	~5
MiSeq	Medium (100k)	1.2k	Low	57 kg	24	13	Medium (26)	~50
MinION	Low (1k)	0.65k	Medium	450 g	1	6	Medium (12)	~50
Flongle	N/A*	0.1k	High**	570 g	12	1	Low (2)	~50

* The flongle is an adaptor that is used on a MinION instrument

** High yield variability on the flongle is likely the consequence of the very recent development of the technology. We expect yield variability to decrease significantly in the future.

*** Portability is listed as a surrogate for ease in utilization of listed instruments, e.g. at outreach clinical centers. Flonge weight is sum of adapter and MinION

**** Nanopore sequencing allows real time calling of sequence data and in the case of a MinION can result in retrieval of copy number profiles in as little as 1 hour

***** Total reads is under assumption of a typical Mid-output NextSeq run and a short molecule MinION run.

****** Costs is calculated by dividing (Cost.reagent.per.run)/(mutiplex.per.run)

Supplementary Figure 1

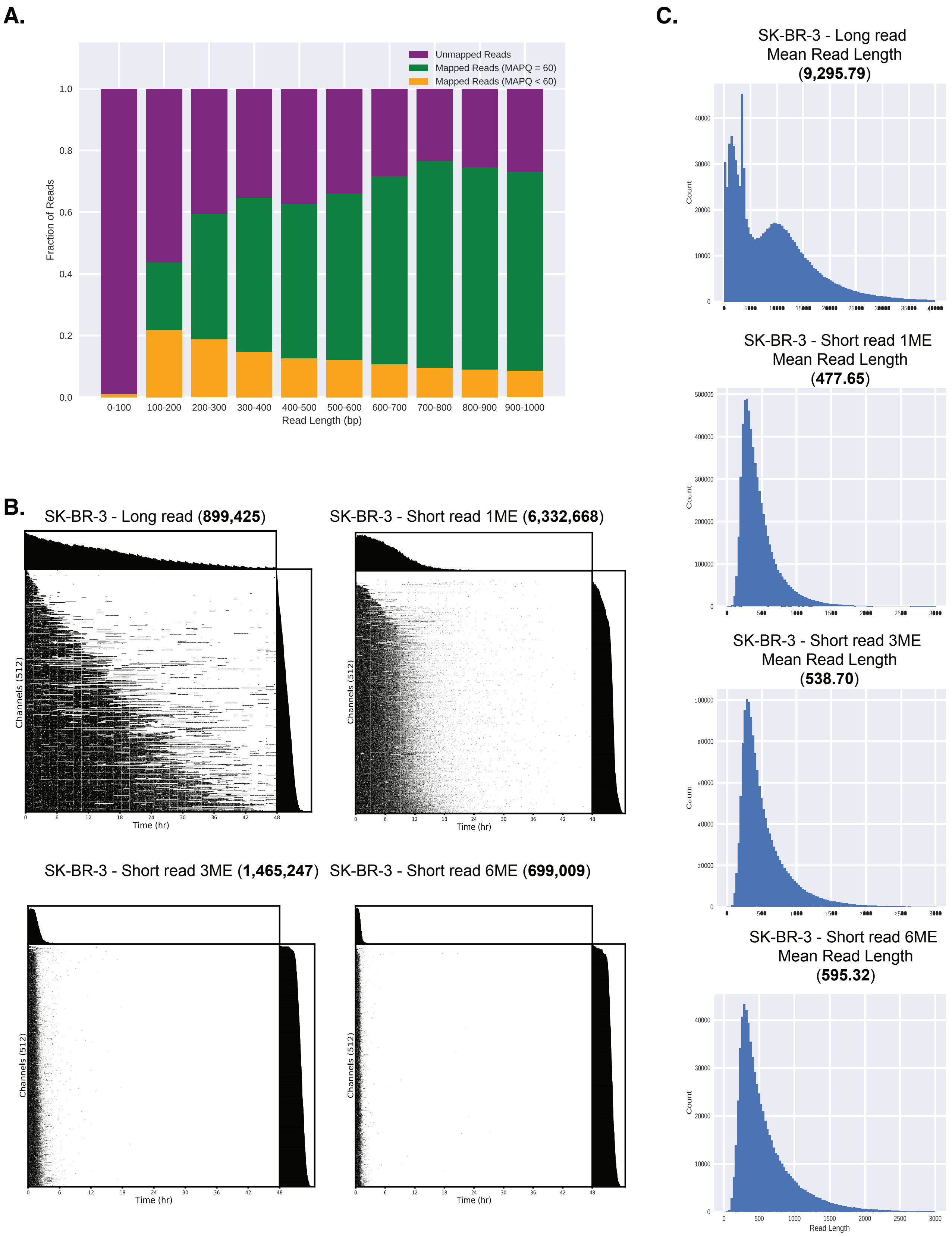

Supplementary Figure 1. Short DNA molecule sequencing on a MinION device yields high number of sequencing reads. (A) Relationship between molecule length and mappabilitty based on simulation analysis (Methods). **(B)** Relationship between nanopore occupancy and read count yields on a MinION device using different SK-BR-3 sequencing library preparations and loading conditions. **(C)** Read length distrubition of the different SK-BR-3 sequencing library preprations and loading conditions as in (A).

Supplementary Figure 2

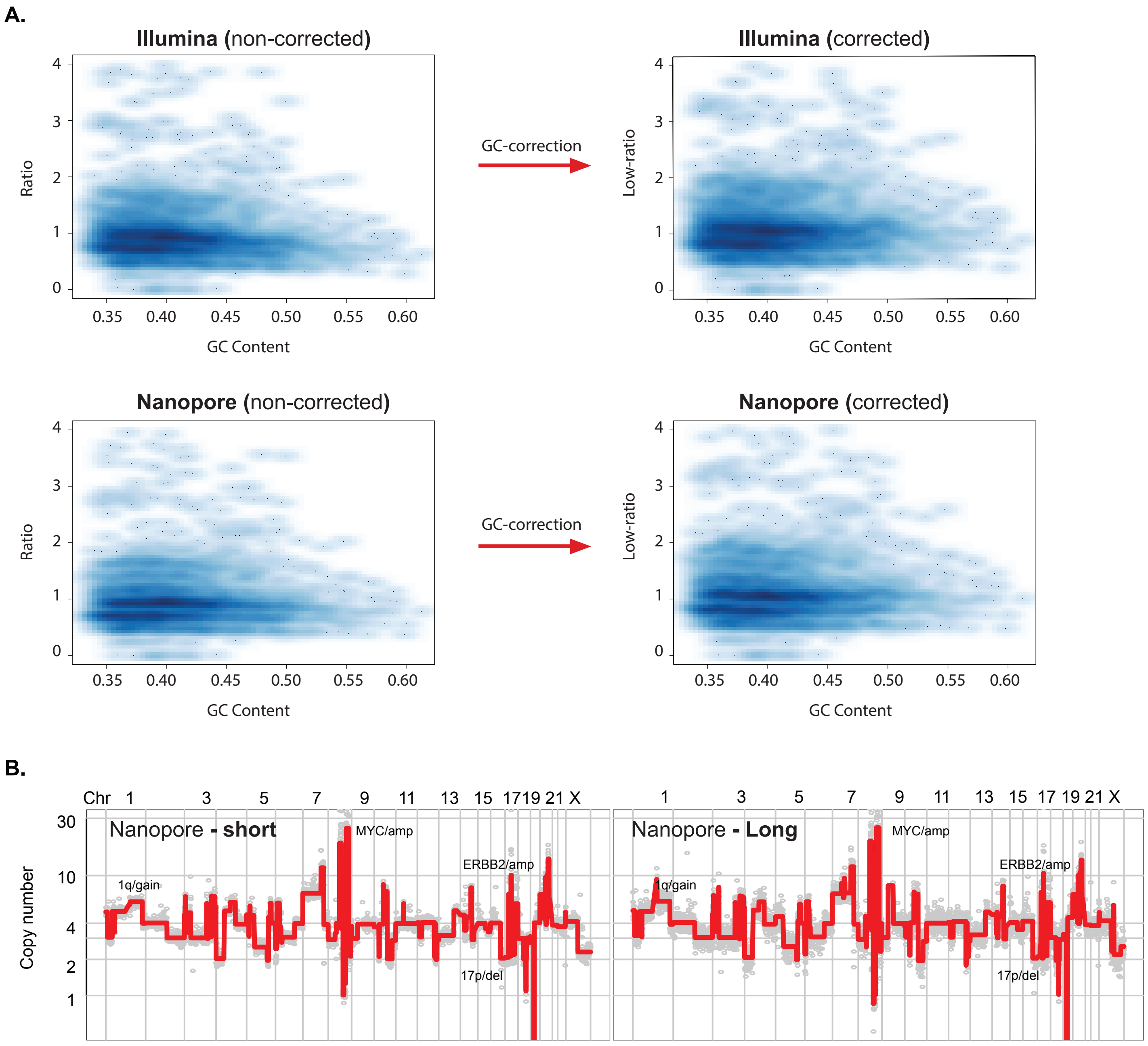

Supplementary Figure 2. Nanopore sequence data normalization and copy number inference in SK-BR-3 datasets. (A) Scatter density plots illustrating GC bias in the SKBR3 Illumina and Short-1ME nanopore data (left panels) and scatter density plots of corrected data using LOWESS smoothing (right panels). **(B)** Genome-wide copy number profiles of SK-BR-3 sequenced using short molecule nanopore (leftpanel) and long molecule nanopore (right panel) data. Profiles at plotted when dividing the genomes in 5 thousand bins (i.e. 5k bins). Examples of detected CNAs are annotated on the profiles.

Supplementary Figure 3

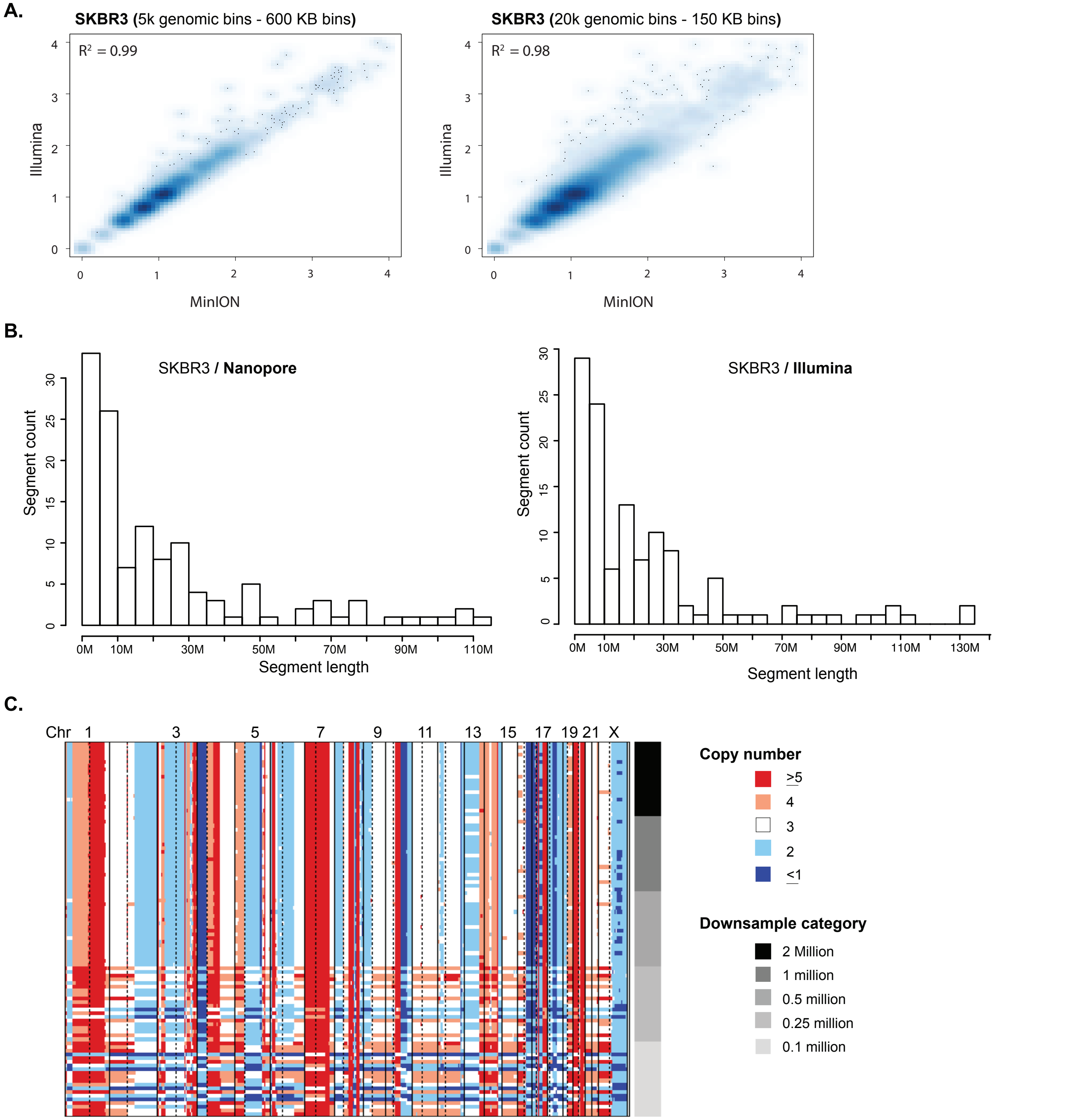

Supplementary Figure 3. MinION short molecule sequencing data returns highly accurate genome-wide copy number information. (A) Scatter density correlation plots of normalized read count values from Illumina and MinION short read (Short 1ME) data. Scatter density plots are illustrated for two resolutions; 5k and 20k (i.e. 5000 genomics bins and 20,000 genomics bins respectively). Pearson correlation values for the analysis at each resolution are provided.
(B) Histogram of the size ofcopy number alterations (in MB) detected in nanopore (left panel) and Illumina (right panel) short molecule sequencing data of SKBR3 cell line. **(C)** Downsampling read simulations of short molecule nanopore sequencing data (1ME)

Supplementary Figure 4

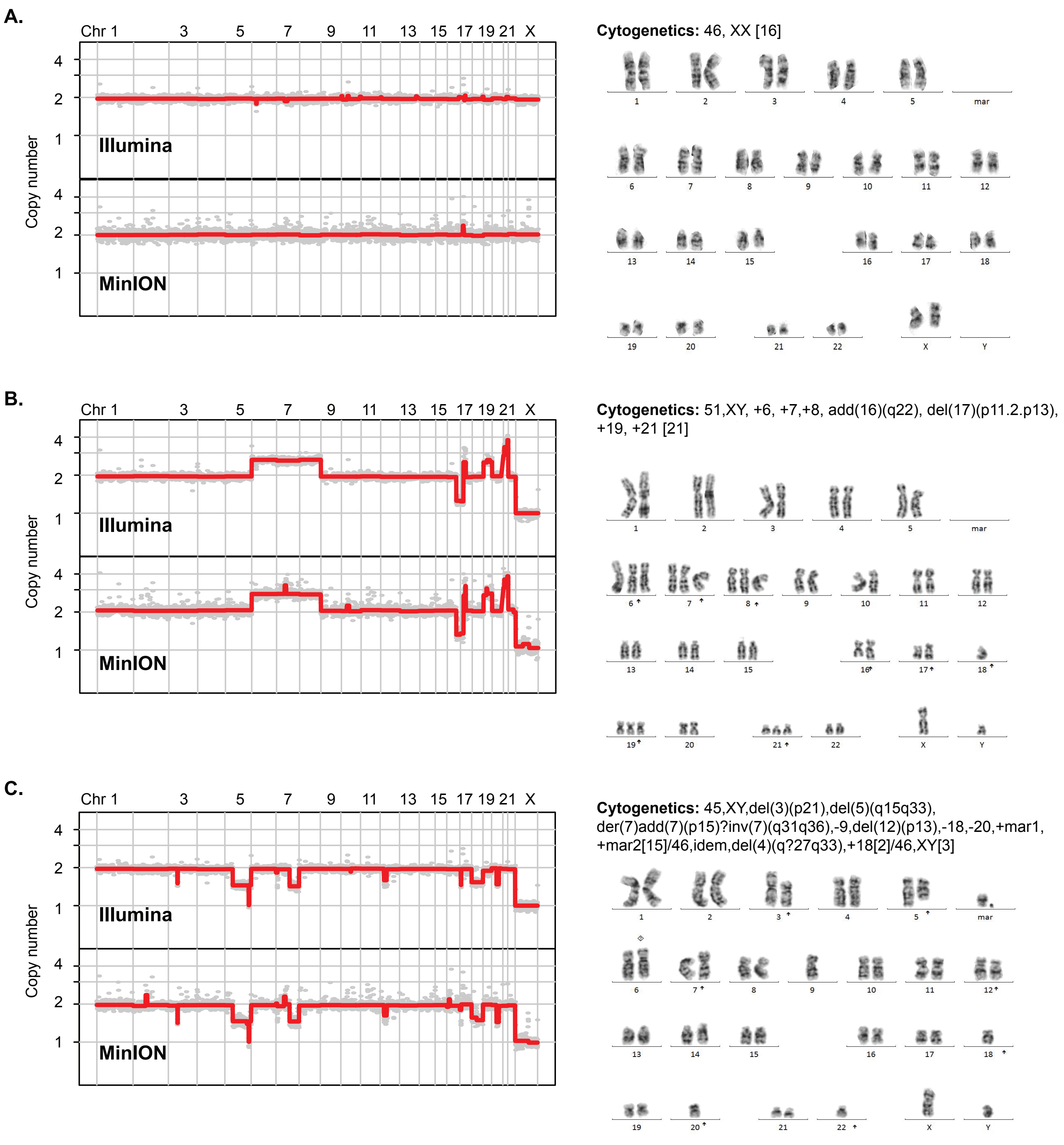

Supplementary Figure 4. Short molecule sequencing on a MinION device reproducibly yields accurate, high resolution copy number information. (A-C) Left panels **-** Genome-wide copy number profiles of ormal and complex karyotype samples sequenced using short read/molecule nanopore sequencing on a MinION (lower panel) and an Illumina (upper-panel) device. Right panels - matching chromosome spread, microscopy images of cytogenetically analyzed AML samples. Cytogenetic annotations are provided on to of the microscopic images.

Supplementary Figure 5

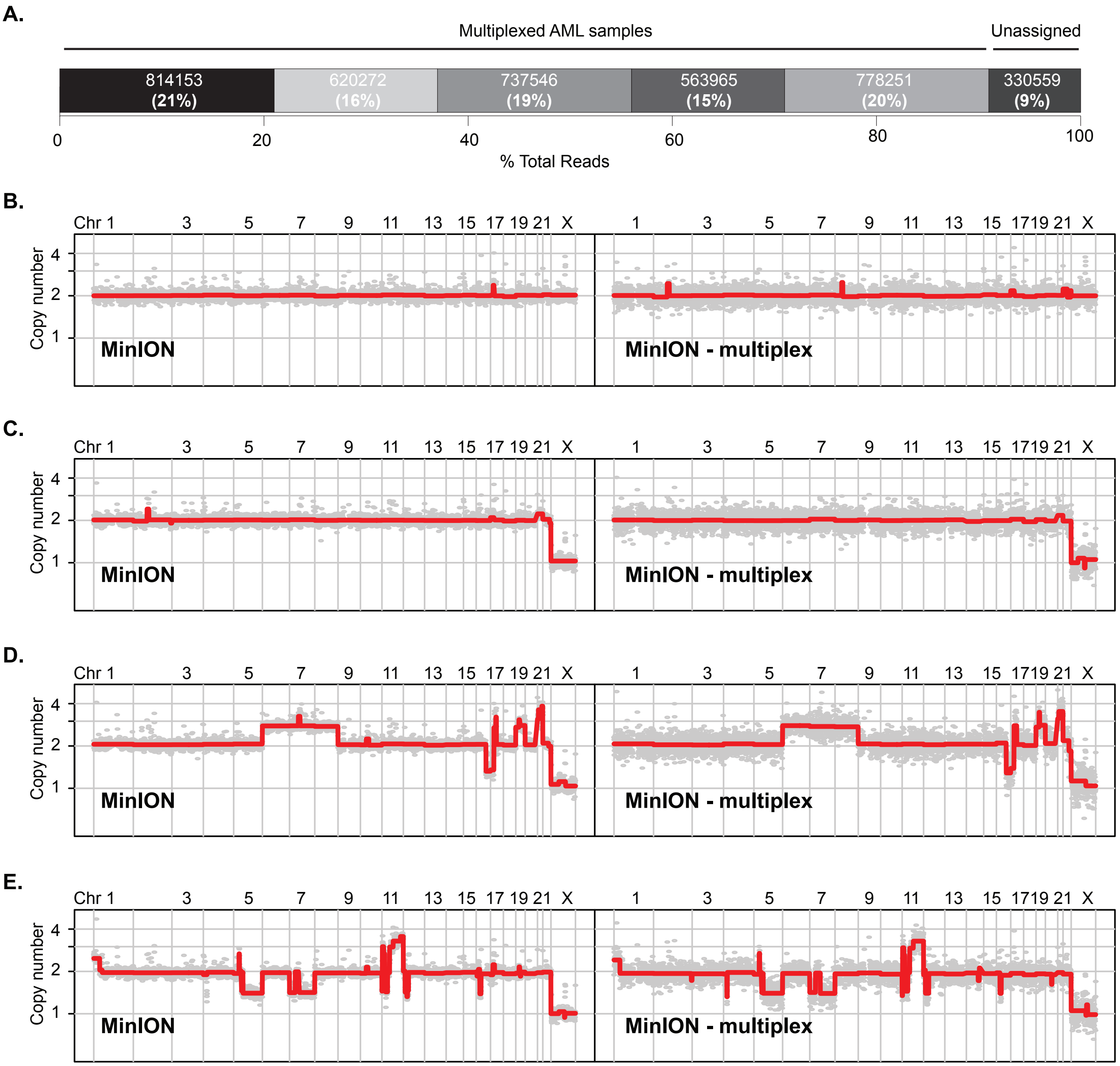

Supplementary Figure 5. Multiplex short molecule sequencing on a MinION device allow accurate and cheap inference of CNAs. (A) Bar plot quantification of de-multiplexed sequencing reads and percentages per pooled, barcoded AML samples plus unassigned reads. **(B-E)** Genome-wide copy number profile of a two normal karyotype (B,C) and two complex karyotype (D, E) samples sequenced on a MinION device in non-multiplex (left panel) and multiplex (right panel) fashion.

Supplementary Figure 6.

A. **C.**

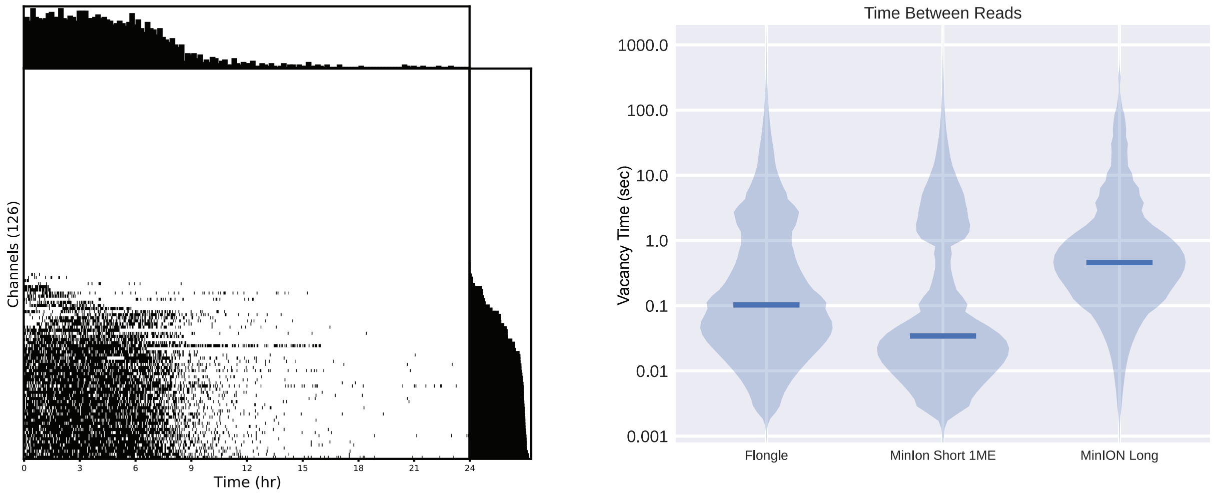

B.

Sample/ ID	Run.Type	Read Count	Yield (Mbp)	Passed (%)	Q-Score Mean	Read- Length Mean	Read N50	Minutes to 250k reads
AML2	MinION	6,263,490	2,886.35	82.82	8.95	460.82	519	15.53
AML3	MinION	3,935,726	1,753.57	86.68	9.29	445.55	488	15.58
AML6	**Flongle**	578,420	257.10	65.36	8.26	444.48	479	167.70
AML7	**Flongle**	296,062	128.04	71.87	8.52	432.49	473	294.52

Supplementary Figure 6. Short molecule sequencing using a Flongle adaptor returns high read counts and allows accurate copy number inference. (A) Channel activity throughout the Flongle run - see Fig1 for detailed explanation of figure. **(B)** Flongle short molecule sequencing metrics compared to MinION runs. **(C)** Distributions of vacancy time (i.e. time between when one molecule finishes and one starts) for Flongle run, compared to the long and short 1ME MinION runs.
